# Molecular Subtyping of *Blastocystis* sp. Isolated from Farmed Animals in Southern Italy

**DOI:** 10.3390/microorganisms9081656

**Published:** 2021-08-03

**Authors:** Simona Gabrielli, Marialetizia Palomba, Federica Furzi, Emanuele Brianti, Gabriella Gaglio, Ettore Napoli, Laura Rinaldi, Renato Aco Alburqueque, Simonetta Mattiucci

**Affiliations:** 1 Department of Public Health and Infectious Diseases, Sapienza University of Rome, Piazzale Aldo Moro 5, 00185 Rome, Italy; simona.gabrielli@uniroma1.it (S.G.); marialetizia.palomba@uniroma1.it (M.P.); federica.furzi@uniroma1.it (F.F.); renatoacoalburqueque@gmail.com (R.A.A.); 2 Diagnostic Parasitology Laboratory, Umberto I University Hospital, Viale del Policlinico 155, 00185 Rome, Italy; 3 Department of Veterinary Sciences, University of Messina, Polo Universitario Annunziata, 98168 Messina, Italy; ebrianti@unime.it (E.B.); ggaglio@unime.it (G.G.); enapoli@unime.it (E.N.); 4 Department of Veterinary Medicine and Animal Production, University of Naples Federico II, Via della Veterinaria 1, 80137 Naples, Italy; lrinaldi@unina.it

**Keywords:** *Blastocystis*, subtypes, animals, Italy, molecular identification, phylogenetic analysis, zoonotic transmission

## Abstract

*Blastocystis* is a common intestinal protist distributed worldwide, infecting humans and a wide range of domestic and wild animals. It exhibits an extensive genetic diversity and, so far, 25 distinct small subunit ribosomal RNA (*SSU* rRNA) lineages termed subtypes (STs)) have been characterized; among them, 12 have thus far been reported in humans. The aims of the present study were to detect and genetically characterize *Blastocystis* sp. in synantropic animals to improve our current knowledge on the distribution and zoonotic transmission of *Blastocystis* STs in Italy. Samples were collected from *N* = 193 farmed animals and submitted to DNA extraction and PCR amplification of the *SSU* rRNA. *Blastocystis* was detected in 60 samples (31.08%) and successfully subtyped. Phylogenetic analysis evidenced that the isolates from fallow deer, goats, and pigs (*N* = 9) clustered within the ST5; those from pheasants (*N* = 2) in the ST6; those from chickens (*N* = 8) in the ST7; those from sheep (*N* = 6) in the ST10; and those from water buffaloes (*N* = 9) in the ST14 clade. The comparison between the present isolates from animals and those previously detected in humans in Italy suggested the animal-to-human *spillover* for ST6 and ST7. The present study represents the widest *Blastocystis* survey performed thus far in farmed animals in Italy. Further epidemiological studies using molecular approaches are required to determine the occurrence and distribution of *Blastocystis* STs in other potential animal reservoirs in Italy and to define the pathways of zoonotic transmission.

## 1. Introduction

*Blastocystis* is a common intestinal protist distributed worldwide, currently known as belonging to the Stramenopiles [1], infecting humans and a wide range of domestic and wild animals [2]. As the pathogenetic role is still controversial, there is not yet a consensus about the term to describe the host–protist interaction, which could be considered an “infection” or a “colonization” [3].

The life cycle of *Blastocystis* remains incompletely known thus far. However, experimental infectivity studies in animals have demonstrated that the water- and environmental-resistant infective cysts are the transmission stage [4,5]. Upon ingestion, excystment takes place in the host intestine, giving rise to vacuolar forms, which divide by binary fission and may develop further into amoeboid or granular forms. Later, encystment may occur during the passage down the colon before cyst excretion in the feces [6].

Molecular studies based on the small subunit ribosomal RNA (*SSU* rRNA) have so far allowed the identification in mammalian and avian hosts of possibly 25 distinct ribosomal lineages, termed subtypes (STs), which could be considered separate species [7,8]. Among the *Blastocystis* ribosomal lineages, 12 subtypes have thus far been identified in humans. In detail, STs 1–4 have been frequently found in humans, but they have also been detected in hoofed mammals, primates, pigs, cattle, rodents, and even birds [9]. Conversely, STs 5–8 have been rarely reported in humans and more commonly found in animals. For instance, ST5 is typically detected in hoofed animals, ST6 and ST7 in birds, and ST8 in non-human primates [10].

Therefore, it has been proposed that a portion of human infection or colonization by *Blastocystis* may result from the zoonotic transmission of the protist. However, the contribution of animal sources to human infection/colonization remains to be confirmed, as the direction of the transmission route to humans is still uncertain [11].

In recent years, the presence of *Blastocystis* has also attracted attention in Italy, where few epidemiological surveys have been published so far, demonstrating the occurrence in humans of seven STs, including those considered as zoonotic (i.e., ST1, ST2, ST3, ST4, ST6, ST7, and ST8) [12,13,14,15,16].

In addition, the protist has been identified in fresh products [17] and in untreated drinking water [18], providing evidence of transmission thought contaminated food or water.

Despite that a prevalence rate of about 7% has been reported in the Italian population [15,19], along with the identification of zoonotic STs in humans [12,13,14], studies investigating the animal species harboring the protist in Italy and their potential role as a source of human infection/colonization still remain limited. So far, the detection of different STs from animals was carried out in few animal categories such as zoo mammals [20], dogs [21], imported macaques [22], and domestic and wild suids [23].

In this frame, the aims of the present study were to (i) detect and genetically characterize *Blastocystis* in synanthropic animals such as farmed animals and (ii) improve our current knowledge on the distribution of *Blastocystis* STs in animals in Italy and their possible transmission routes to humans.

## 2. Materials and Methods

### 2.1. Sample Collection

During the year 2018, fecal samples were randomly collected from animals sampled from different farms in two regions of Southern Italy, which were selected because of the simultaneous presence of different animal species including poultries and untreated with antibiotics in the previous months. In detail, three farms were located in the Messina province (Sicily region), while samples from water buffaloes were collected in nine and two farms from the Salerno and Caserta province (Campania region), respectively.

Samples were directly collected from rectal ampulla to avoid environmental contamination. Animals included in the study were apparently in a good state of health and did not report any clinical signs of concurrent infection by other pathogens.

### 2.2. DNA Extraction and Sequencing of Blastocystis Isolates

Genomic DNA was extracted from each fecal sample using the Fecal DNA kit (Bioline, Taunton, MA, USA) according to the manufacturer’s protocol. A fragment of about 500 bp from the *SSU* rRNA gene was amplified using the primers Blast 505–532 (5′-GGAGGTAGTGAC AATAAATC-3′) and reverse Blast 998–1017 (5′-TGCTTTCGCACTTGTTCATC-3′) following the protocol proposed by Santín et al. [24]. Positive samples were further amplified using the primers RD5 (5′-ATCTGGTTGATCCTGCCAGT-3′) and BhRDr (5′-GAGCTTTTTAACTGCAACAACG-3′), in order to compare sequences obtained in this study with human isolates from our previous survey [13,15], using the PCR-conditions described in Scicluna et al. [25]. PCR amplicons obtained with this PCR protocol were purified and Sanger sequenced from both strands with the same primers, at the Sequencing Service of Biofab Research through an Automated Capillary Electrophoresis Sequencer ABI3730 DNA Analyzer (Applied Biosystems, Carlsbad, CA, USA), using the BigDye^®^ Terminator v3.1 Cycle Sequencing Kit (Life Technologies, Carlsbad, CA, USA).

The resulting chromatograms were analyzed and edited in the computer software Chromas version 2.33 (South Brisbane, Queensland, Australia). The sequences obtained were then compared to the sequences of *Blastocystis* STs, previously deposited in GenBank™ (https://www.ncbi.nlm.nih.gov/genbank/, accessed on 1 February 2021) and PUBMLST databases (https://pubmlst.org/organisms/blastocystis-spp, accessed on 1 February 2021). The subtypes (STs) were identified by determining the exact match (100%) or closest identity (99%), according to the classification of the subtypes given by Stensvold et al. [26].

All sequences were submitted to GenBank™, reported with their accession numbers MZ242080–MZ242085.

### 2.3. Phylogenetic and Genetic Diversity Analyses

Phylogenetic analysis, based on the obtained sequences at the *SSU* rRNA from those positive samples, was inferred from Bayesian inference (BI) using MrBayes, v. 3.2.7 [27]. The Bayesian posterior probability analysis was performed using the MCMC algorithm, with four chains, 0.2 as the temperature of heated chains, 5,000,000 generations, a subsampling frequency of 500, and a burn-in fraction of 0.25. Posterior probabilities were estimated and used to assess the support for each branch. Values with a 0.90 posterior probability were considered well-supported. Homologous *Blastocystis SSU* rRNA gene nucleotide sequences, available in GenBank database (accessed on 1 February 2021), were included to generate the phylogenetic tree, rooted using *Proteromonas lacertae* a.n. U37106.1 as outgroup.

Genetic distances were computed using the Kimura 2-Parameters (K2P) model [28] with 1000 bootstrap re-samplings, by MEGA Software, version 7.0.

## 3. Results

A total of *N* = 193 fecal samples were collected from farmed animals, i.e., water buffaloes (*N* = 101), cow (*N* = 13), donkey (*N* = 2), duck (*N* = 2), fallow deer (*N* = 1), goat (*N* = 9), chicken (*N* = 17), horse (*N* = 3), ostrich (*N* = 2), peacock (*N* = 7), pheasant (*N* = 11), pig (*N* = 13), sheep (*N* = 11), and turkey (*N* = 1). Out of 193 stool samples, 60 (31.08%) were positive at *Blastocystis* by the molecular analysis. The number of positive samples varied among the animal host species, as detailed in Table 1.

Among the positive samples, 34 were successfully sequenced, showing the presence of a single ST. The remaining 26 PCR products displayed unreadable sequences for the entire region (about 600 bp). Only sequences that showed chromatograms with single peaks were analyzed, while the subtype identification was not performed from chromatograms with double signals. Indeed, the presence of double signals in the chromatograms, which is very common in *Blastocystis* sequences from animals, may be indicative of mixed infections by different STs in the same host. In this case, the cloning of the PCR product or next generation amplicon sequencing would be required to discriminate such different subtypes [8]. In this study, the ST identification protocol followed the PCR amplification coupled with sequencing, which preferentially allowed to identify the predominant subtype.

**Figure 1:**
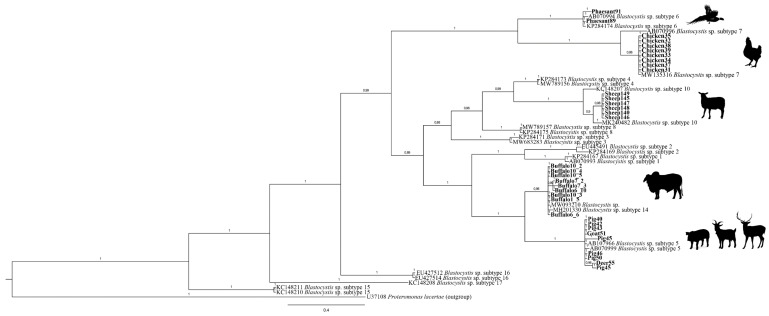
Bayesian inference (BI) obtained from *SSU* rRNA sequences data set of *Blastocystis* from animals analyzed in this study, and those from STs previously deposited and available in GenBank™, performed by MrBayes, v. 3.2.7, using the GTR + I + G substitution model. *Proteromonas lacertae* was used as outgroup. The sequences obtained in this study are given in bold. Taxon labels consist of GenBank number followed by subtype name. Icons indicate where the specimens were collected.

Analysis of readable sequences showed a high identity (99–100%) to homologous sequences of *Blastocystis* previously reported in GenBank™, allowing the BLAST identification of five distinct STs (i.e., ST5, ST6, ST7, ST10, and ST14) (Table 2). The topology of Bayesian inference (BI) showed that the *Blastocystis* isolates analyzed here clustered, with high probability value (from 99% to 100%), in five distinct clades. In particular, the sequences of *Blastocystis* obtained from deer (*n* = 1), pig (*n* = 7), and goat (*n* = 1) clustered in the clade that includes the previously deposited sequences of the subtype ST5; those from pheasant (*n* = 2) and chicken (*n* = 8) were clustered in the clades formed by the available sequences of ST6 and ST7, respectively; and, finally, those from sheep (*n* = 6) and water buffalo (*n* = 9) were included with high probability value (100%), respectively, in the clades formed by the reference sequences of the subtypes named ST10 and ST14 (Figure 1).

Alleles named using the *Blastocystis* database (http://pubmlst.org/Blastocystis/, accessed on 1 February 2021) allowed the detection of three distinct alleles (i.e., allele 17, 115, and 153) within the ST5 found here, from fallow deer, pig, and goat hosts, respectively. Allele 157 was observed in the isolates ST14 from the water buffaloes, while alleles 122, 41, and 152 were identified within the ST6, ST7, and ST10, isolated from chicken, pheasant, and sheep, respectively (Table 1). In addition, the isolates ST6 and ST7 detected in pheasant and chicken in the present study showed a high similarity (99–100%, respectively) with the ST6 and ST7 sequences subtyped from the human host in Italy [13,15].

## 4. Discussion

To the best of our knowledge, this study represents the widest survey on *Blastocystis* STs in farmed animals performed so far in Italy, including a total of 14 different animal species. The study evidences the occurrence of the protist in about 30% of the analyzed livestock, showing the circulation among them of five *Blastocystis* STs genetically identified, i.e., ST5, ST6, ST7, ST10, and ST14. These findings improve the current knowledge on the animal species harboring *Blastocystis* in Italy and support the role of these animal species as potential sources of human infections. Indeed, the comparison of sequences from animals to those from human isolates, such as ST6 and ST7, evidenced a high similarity (99–100%) among animal and human isolates identified in this and our previous study, respectively [13,14,15]. Accordingly, allele analysis has shown the occurrence of the same alleles (i.e., 122 and 41) that have been found in ST6 and ST7, respectively, isolated from both humans [13,15] and animals (present study), suggesting that poultry may be a source of human infection.

We cannot depict the precise mode of circulation between animals and humans of those STs; however, we could hypothesize that it may happen through contaminated water with animal stools [6]. Indeed, *Blastocystis* has been reported as a neglected waterborne protist and recently found in drinking water [18,30], a drinking water treatment plant [31], and ready-to-eat packaged salads [17]. However, the identification of zoonotic STs and alleles in edible animals such as poultry suggests the potential risk of *Blastocystis* transmission also by human handling of those farmed animals, as well as through the consumption of products of animal origin (e.g., eggs). Further investigation aimed to detect *Blastocystis* in different food matrices may contribute to clarifying the source of infection and patterns of transmission to humans of such zoonotic STs in Italy.

In recent years, several studies willing to address the issue of *Blastocystis* pathogenicity in humans suggested that it could be related to genetic differences of distinct STs [32,33], correlating with the ST1, ST4, and ST7 with pathological alterations in humans, while ST2 and ST3 have been identified as non-pathogenic [33,34,35]. A recent multi-locus sequence typing analysis of *Blastocystis* ST3 and ST4 has provided valuable insight into genetic variation within and between the two subtypes, evidencing a high or low level of genetic diversity at the intra-subtype level in ST3 and ST4, respectively [35]. Similar results were obtained in our previous survey from Italian patients [13] where three haplotypes (H1, H3, and H7) have been identified in ST3 isolates, while a single haplotype (H2) was observed in ST4 symptomatic patients. Thus, we suggested that intra-subtype diversity shown by ST3 and ST4 could be linked to the evolutionary history of *Blastocystis* subtypes, and that ST3 may have co-evolved with human hosts over a longer period than that experimented by ST4. This latter, instead, may more recently have had a *spillover* from rodents to humans; therefore, it shows a low level of genetic variability. According to the hypothesis of the recent evolutionary history of the ST4, a higher pathogenicity in humans was observed as due to ST4, with respect to other subtypes, found to be associated in patients with IBS, IBD, or chronic diarrhea [13].

In this study, similar consideration may be extended to the ST7, which has been reported to be strongly associated with gastrointestinal symptoms in humans and showed pathogenic properties, not observed in other STs, as well as an extensive intra- and inter-subtype variability in citopathogenicity [33,36]. According to our experience, features of gastro-intestinal symptoms were also found in human patients harboring ST7, confirming its pathogenetic role (Mattiucci and Gabrielli, personal communication). Conversely, the identification of allele 41 observed in ST7 isolates from both humans and animals in Italy seems to suggest the existence of a low level of intra-subtype genetic variability, in contrast with that previous reported [33]. However, this anecdotic finding should be confirmed by genetic investigation carried out on several other samples corresponding to ST7 from both animals and humans. Furtherly, molecular epidemiology carried out simultaneously on fecal samples collected from humans and animals sharing farm enclosures and environmental characteristics will help to clarify the circulation of the same *Blastocystis* STs and alleles among those hosts.

Despite that the present study represents the widest *Blastocystis* survey performed thus far in farmed animals in Italy, some limitations related to the fact that few samples from some animal species (i.e., from fallow deer or from turkey) were collected should be taken into account. As a consequence, any conclusive consideration about the possible association between the occurrence of certain subtypes in some animal species or the lack of *Blastocystis* in some others cannot be definitively drawn. Further, as the epidemiological scenario described here was restricted to a few Italian regions, further surveys should be planned to include other animal species and more samples from the above tested animals, as well as from other geographical areas of Italy. In addition, because possible multiple co-infection by distinct STs would occur in the same farmed animal, an RT-PCR protocol would be developed based on the melting profiles in order to simultaneously identify different subtypes, or alternatively the cloning protocol, such as that proposed in other studies [8], would be adopted in future epidemiological studies. Another critical issue concerns the amplicons (26 out 60) that were positive with the primers proposed by Santín et al., 2011 [24] and with those described in Scicluna et al. [25], but they were not included in the phylogenetic analysis as we did not obtain readable products for the entire region (about 600 bp) analyzed with the BI. Despite that we cannot totally exclude that some of these amplicons could be the product of unspecific amplifications, all 26 amplicons were included in the total of positive samples as they were amplified with two protocols described for the identification of *Blastocystis* [24,25].

Moreover, the sampling was devoted to a particular group of animals, which were also selected for their possible role in the transmission of the protist also by derived food product such as eggs. Therefore, further parasitological and molecular investigations need to be carried out with the aim to evaluate other potential animal reservoirs of *Blastocystis* in Italy and to investigate the possible food-borne transmission of the protist.

## 5. Conclusions

The outcome of this study may be considered as a starting point to define the distribution of *Blastocystis* STs in different animal hosts in Italy and to hypothesize pathways of the zoonotic transmission of the protist.

Thus, it becomes evident that a greater knowledge of the evolutionary history of *Blastocystis* STs could, in future, explain any pathogenic aspects related to distinct subtypes. Studies on the genetic variation within and among identified subtypes may elucidate possible co-evolutionary aspects, and provide data for understanding *Blastocystis* biology, host–parasite interaction, and pathogenicity to humans.

Lastly, as *Blastocystis* has been reported as a neglected waterborne protist, further investigations are needed to identify other food matrices and vehicles as sources of transmission to human and animal hosts in Italy.

In conclusion, these findings represent only the tip of the iceberg concerning the epidemiology of *Blastocystis* in Italy and suggest the need to apply molecular analysis to demonstrate the transmission dynamics among humans, animals, and the environment of this so far debated plastic protist.

## Figures and Tables

**Table 1:** Molecular identification and subtyping of *Blastocystis* collected from various animal species.

Host	Scientific Name	Collected Samples (*N*)	Positive Samples (%)	STs Identified	Alleles
Cow	*Bos taurus*	13	1 (7.69)	untypable	-
Donkey	*Equus asinus*	2	0	-	-
Duck	*Anas* *platyrhynchos domesticus*	2	0	-	-
Fallow deer	*Dama dama*	1	1 (100)	5	17
Goat	*Capra aegagrus hircus*	9	4 (44.4)	5	-
Chicken	*Gallus gallus* *domesticus*	17	8 (47)	7	41
Horse	*Equus caballus*	3	0	-	-
Ostrich	*Struthio camelus*	2	0	-	-
Peacock	*Pavo* sp.	7	3 (42.85)	untypable	-
Pheasant	*Phasianus* *colchicus*	11	6 (54.5)	6	122
Pig	*Sus scrofa* *domesticus*	13	10 (76.92)	5	17, 115, 153
Sheep	*Ovis aries*	11	9 (81.8)	10	152
Turkey	*Meleagris* *gallopavo*	1	0	-	-
Water buffalo	*Bubalus bubalis*	101	18 (17.82)	14	157
**Total**		**193**	**60 (31.08%)**		

**Table 2:** BLAST correspondence of *Blastocystis* sequences obtained in the present study with those *Blastocystis* subtypes (STs) available in the GenBank™ database reported with their host, geographic origin, and accession number.

Sample ID/Isolate	*Blastocystis* Subtype (GenBank Accession Number)	Host	Locality	References	Similarity (%)
Phaesant91	ST6 (MW713074)	Tibetan goat	China	Chang et al., unpublished	99.63
Phaesant89	ST6 (MW713074)	Tibetan goat	China	Chang et al., unpublished	99
Chicken31	ST7 (KY488585)	-	Southwest of Iran	Salehi et al., unpublished	100
Chicken32	ST7 (KY488585)	-	Southwest of Iran	Salehi et al., unpublished	100
Chicken33	ST7 (KY488585)	-	Southwest of Iran	Salehi et al., unpublished	100
Chicken34	ST7 (KY488585)	-	Southwest of Iran	Salehi et al., unpublished	100
Chicken35	ST7 (KY488585)	-	Southwest of Iran	Salehi et al., unpublished	100
Chicken37	ST7 (KY488585)	-	Southwest of Iran	Salehi et al., unpublished	100
Chicken38	ST7 (KY488585)	-	Southwest of Iran	Salehi et al., unpublished	100
Chicken39	ST7 (KY488585)	-	Southwest of Iran	Salehi et al., unpublished	100
Sheep140	ST10 (MF186708)	Wallaby	-	Betts et al., unpublished	99.80
Sheep145	ST10 (MF186708)	Wallaby	-	Betts et al., unpublished	99.80
Sheep146	ST10 (MF186708)	Wallaby	-	Betts et al., unpublished	99.80
Sheep147	ST10 (MF186708)	Wallaby	-	Betts et al., unpublished	99.80
Sheep148	ST10 (MF186708)	Wallaby	-	Betts et al., unpublished	99.80
Sheep149	ST10 (MF186708)	Wallaby	-	Betts et al., unpublished	99.80
Buffalo10_2	ST14 (MW682196)	Goat	Poland	Rudzinska, unpublished	99.08
Buffalo10_4	ST14 (MW682196)	Goat	Poland	Rudzinska, unpublished	99.08
Buffalo10_5	ST14 (MW682196)	Goat	Poland	Rudzinska, unpublished	99.08
Buffalo7_2	ST14 (MW682196)	Goat	Poland	Rudzinska, unpublished	98.90
Buffalo7_3	ST14 (MW682196)	Goat	Poland	Rudzinska, unpublished	98.72
Buffalo6_10	ST14 (MW682196)	Goat	Poland	Rudzinska, unpublished	98.72
Buffalo10_3	ST14 (MW682196)	Goat	Poland	Rudzinska, unpublished	98.75
Buffalo1_5	ST14 (MW682196)	Goat	Poland	Rudzinska, unpublished	99.08
Buffalo6_6	ST14 (MW682196)	Goat	Poland	Rudzinska, unpublished	99.08
Pig40	ST5 (MK801418)	Pig	Romania	Wylezichet al., 2019 [29]	100
Pig42	ST5 (MN493729)	Wild boar	South Korea	Lee and Kwak, unpublished	100
Pig43	ST5 (MN493729)	Wild boar	South Korea	Lee and Kwak, unpublished	100
Pig45	ST5 (MN493729)	Wild boar	South Korea	Lee and Kwak, unpublished	100
Pig46	ST5 (MN493729)	Wild boar	South Korea	Lee and Kwak, unpublished	100
Pig50	ST5 (MN493729)	Wild boar	South Korea	Lee and Kwak, unpublished	100
Goat51	ST5 (MN493729)	Wild boar	South Korea	Lee and Kwak, unpublished	100
Deer55	ST5 (MK801418)	Pig	Romania	Wylezichet al., 2019 [29]	100
